# Metabolic syndrome and spatial disparities: The role of socioeconomic deprivation and medical resource availability in the Cijin district, Taiwan

**DOI:** 10.1002/kjm2.12908

**Published:** 2024-11-14

**Authors:** Pei‐Hung Su, Jong‐Rung Tsai, Wei‐Lun Chang, Hui‐Min Hsieh

**Affiliations:** ^1^ Kaohsiung Municipal Ci‐Jin Hospital, Kaohsiung Medical University Hospital Kaohsiung Medical University; ^2^ Department of Business Management, Ph.D. Program of Health Care Management National Sun Yat‐sen University Kaohsiung Taiwan; ^3^ Department of Respiratory Therapy, College of Medicine Kaohsiung Medical University Kaohsiung Taiwan; ^4^ Department of Internal Medicine, Kaohsiung Medical University Hospital Kaohsiung Medical University Kaohsiung Taiwan; ^5^ Department of Internal Medicine Kaohsiung Municipal Ci‐Jin Hospital Kaohsiung Taiwan; ^6^ Department of Public Health Kaohsiung Medical University Kaohsiung Taiwan; ^7^ Department of Medical Research Kaohsiung Medical University Hospital Kaohsiung Taiwan; ^8^ Department of Community Medicine Kaohsiung Medical University Hospital Kaohsiung Taiwan; ^9^ Center for Big Data Research Kaohsiung Medical University Kaohsiung Taiwan; ^10^ Research Center for Environmental Medicine Kaohsiung Medical University Kaohsiung Taiwan

**Keywords:** health inequality, medical resource availability, metabolic syndrome, socioeconomic area deprivation

## Abstract

Metabolic syndrome (MetS) is a global health concern with spatial disparities, especially in disadvantaged neighborhoods. This study aimed to examine the association between area‐level socioeconomic deprivation, the availability of medical resources in disadvantaged areas such as the Cijin district, and the prevalence of MetS in Taiwan. We used two representative secondary cross‐sectional datasets, including physical examinations and lifestyle surveys from 2016 to 2020, sourced from the Taiwan Biobank and the Cijin District Adult Lifestyle and Health Survey. Our findings indicate that residing in the Cijin district, characterized by socioeconomic deprivation and limited medical resources, is associated with significantly higher odds of MetS (aOR = 1.45, 95% CI = 1.28–1.64, *p* <0.001). Additionally, living in areas with medium (aOR = 1.12, 95% CI = 1.07–1.17, *p* <0.001) and high area‐level socioeconomic deprivation indexes (aOR = 1.13, 95% CI = 1.01–1.25, *p* <0.001) is linked to a higher likelihood of MetS. Conversely, residing in high medical resource availability index areas is associated with a lower risk of MetS (aOR = 0.92, 95% CI = 0.86–0.99, *p* = 0.026). We found a link between socioeconomic deprivation and limited medical resources, especially in disadvantaged areas such as the Cijin district, contributing to a higher MetS risk. Residents in these areas often struggle to access healthcare and preventive care. Addressing these disparities requires comprehensive public health initiatives and targeted policy interventions to improve residents' well‐being.

## 1 BACKGROUND

Metabolic syndrome (MetS) is a globally recognized problem, with estimated prevalence worldwide of approximately 20%–5%. When compared across regions, it was estimated to increase from 37.6% in 2011 to 41.8% in 2018 in the United States.^1^, ^2^ In Taiwan, the estimated prevalence of MetS in adults increased from 22.5% in 2008 to 34.6% in 2020.^3^ MetS is diagnosed when an individual fulfills three or more criteria, including “central obesity, elevated blood pressure, hyperglycemia, hypertriglyceridemia, and reduced high‐density lipoprotein cholesterol, that are associated with the development of type 2 diabetes mellitus and cardiovascular disease,” which were defined in previous study.^4^ It is a group of interrelated risk factors that significantly increase the risk of several chronic diseases, leading to an elevated mortality rate.^5^, ^6^

Socioeconomic deprivation is a key factor that creates a stressful environment that triggers and perpetuates metabolic abnormalities through both direct (e.g., poor access to healthy food) and indirect (e.g., chronic stress) pathways.^7^, ^8^, ^9^, ^10^ As Townsend (1987) argued, deprivation is a “condition of visible and demonstrable disadvantage concerning the local community or the broader society to which an individual, family, or group belongs.”^11^ Socioeconomic deprivation acts as a social determinant of health, where the environment and living conditions significantly influence metabolic health outcomes within specific geographic areas, such as neighborhoods or regions.^12^ However, prior research examined the association between neighborhood deprivation and the occurrence of several chronic diseases, with less emphasis on investigating MetS specifically.^13^, ^14^, ^15^, ^16^, ^17^

Resource allocation disparities in underserved areas may reinforce existing socioeconomic inequities.^18^ Given that government services and healthcare access are typically concentrated in urban areas, underserved regions (such as rural or geographically isolated areas) often present challenges due to logistical difficulties, which frequently results in lower priority for resource allocation. Improved population health can be achieved by reducing disparities in the allocation of health resources across regions and addressing differences among social groups. Existing literature has yielded mixed results when exploring the relationships between the distribution of medical resources and health outcomes.^18^, ^19^, ^20^ For instance, Farahani, Subramanian, and Canning (2009) found that increasing the number of physicians by one per 1000 population reduced infant mortality rates, using 99 counties' health system data at 5‐year intervals from 1960 to 2000. In contrast, a pivotal study by Newhouse and Friedlander (1980) used data from the Framingham Heart Study to investigate the influence of healthcare resources on health status in the United States. They evaluated physiological markers, including hypertension prevalence, high serum cholesterol, abnormal electrocardiogram results, and chest x‐rays, rather than mortality or morbidity. Their results echoed those of other US studies that centered on mortality and morbidity, suggesting that increased healthcare resources had limited impact on overall health outcomes.^18^ However, a scarcity remains of empirical research that delves into the influence of region‐specific healthcare resources on MetS as an indicator of health.

This study aimed to examine the association between socioeconomic area deprivation, the availability of medical resources, and specific, relatively disadvantaged areas (such as the Cijin district), and the prevalence of MetS in Taiwan. In light of the prevailing emphasis on depicting the average regional effects in previous studies, there is a conspicuous research gap with regard to examining specific and relatively disadvantaged areas, as opposed to the broader regional perspective. The Cijin District, an urban district within Kaohsiung City in Taiwan, is an isolated sandbar island connected to the city only by ferries and the cross‐harbor tunnel, resulting in limited transportation options. Consequently, compared to other districts in Kaohsiung City, the Cijin district is characterized by relatively inadequate healthcare resources, a higher proportion of elderly residents, and more pronounced socioeconomic deprivation. These factors underscore the need for special attention to this region.^21^ Specifically, we employed two representative secondary cross‐sectional datasets, comprising physical examination and lifestyle surveys conducted from 2016 to 2020 in Taiwan, to investigate the associations between socioeconomic area deprivation, the presence of medical resources in specific and relatively disadvantaged areas (the Cijin district), and other biological factors, with the prevalence of MetS.

## 2 METHODS

### 2.1 Study design and data source

This study used two representative secondary cross‐sectional physical examination and lifestyle survey datasets within 5 years from 2016 to 2020. The first was the baseline data collected through questionnaires, physical examination, and blood and urine tests in the Taiwan biobank datasets (TWB). In 2012, Taiwan launched the TWB, which is government‐supported, and employed a community‐based approach to prospectively collect lifestyle behaviors; environmental risk factors; family histories of common, complex diseases; laboratory tests; and genetic information from individuals aged older than 20 years. As of August 2021, 151,406 participants had enrolled in the TWB.^22^ The second was data source was the Cijin District Adult Life Style and Health Survey Study, which also employed a community‐based approach, and began collecting similar information since 2016 from Kaohsiung Municipal Cijin Hospital and Kaohsiung Medical University for residents living in the Cijin district aged older than 20 years. As of the end of 2021, 1866 participants had enrolled in the Cijin study. This study was approved to use Taiwan biobank data from Academia Sinica (TWBR11103‐01) and received ethical approval from the Institutional Review Board (KMUHIRB‐E(I)‐20210300, KMUHIRB‐SV(I)‐20210082, and KMUHIRB‐G(I)‐20160025). The database application was approved by the Taiwan biobank, Academia Sinica through its repository, and Kaohsiung Municipal Cijin Hospital. All data for TWB and the Cijin study participants is available upon application for research purposes. A detailed of the application process of TWB and Cijin data can be found at https://taiwanview.twbiobank.org.tw/data_appl, and https://www.kmuh.org.tw/web/kmuhdept/6800.

A third data source was national townships/districts‐level statistical open data from the Ministry of the Interior (https://www.moi.gov.tw/) to generate area‐level socioeconomic deprivation measures listed in Table 1, including district population statistics in 2010 and 2020; agriculture, forestry, fisheries, and animal husbandry census statistics in 2010; low‐income household statistics in 2020; population marital status statistics in 2020; and population educational attainment statistics in 2020. A fourth source was national townships/district‐level medical statistical open data from the Ministry of Health and Welfare (https://dep.mohw.gov.tw/) to generate medical resource availability measures, including statistics regarding medical institutions, beds, and certified health professionals in 2020.

**TABLE 1:** Descriptive results of area‐level socioeconomic deprivation and medical resource availability measures in Cijin and nationwide township/districts in Taiwan.

District/townships	Cijin district	Nationwide township /districts^a^
*N*	1	352
Variables	Point estimate^b^	Median (33rd, 67th percentile)
Area‐level socioeconomic deprivation measures		
Proportion of low‐income households	0.40	0.18 (0.15, 0.24)
Proportion of individuals without a high school diploma	0.35	0.28 (0.23, 0.32)
Proportion of divorced and widowed individuals	0.18	0.15 (0.14, 0.17)
Proportion of population aged 65 and older	0.18	0.18 (0.16, 0.20)
Proportion of farming and ranching households	0.05	0.29 (0.16, 0.38)
Area deprivation index (ADI)	0.40	−0.18 (−0.80, 0.45)
Area‐level medical resource availability measures		
Number of acute care hospitals per 10,000 population	0.36	0.00 (0.00, 0.21)
Acute care hospital beds per 10,000 people	7.20	0.00 (0.00, 22.08)
Physicians per 10,000 people	5.04	6.15 (4.16, 10.03)
Primary care clinics per 10,000 people	3.60	5.59 (4.39, 7.18)
Medical resource index (MRI)	−0.38	−0.69 (−0.93, 0.12)

Abbreviations: ADI, area deprivation index; MRI, medical resource index.

^a^ Counties on the offshore islands, such as Kinmen and Matsu counties, were excluded.

^b^ The ADI and MRI measures in the Cijin district were only one‐point estimates. Therefore, there were no low or high ends of the ADI and MRI distributions.

### 2.2 Study population

**FIGURE 1:**
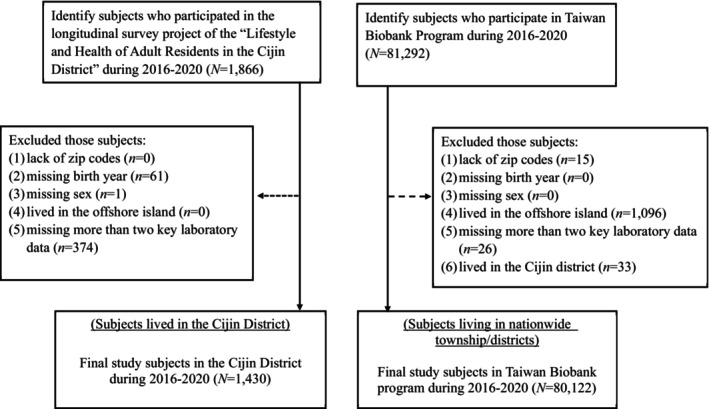
Flow chart of inclusion and exclusion criteria.

We initially identified 81,292 participants in the TWB between 2016 and 2020. We excluded individuals with missing geographic area zip codes, birth year, gender information, and residents of offshore islands (Kinmen and Matsu counties). Additional exclusions applied to those with missing data for more than two laboratory results used to identify MetS. In addition, 33 subjects residing in the Cijin district in the TWB dataset were excluded. Similar inclusion and exclusion criteria were employed to identify study subjects in the Cijin survey study. The study's inclusion and exclusion criteria are presented in Figure 1. Ultimately, the study included a total of 1430 subjects from the Cijin survey study and 80,122 subjects from the TWB.

### 2.3 Variable definition

#### 2.3.1 Prevalent risk of MetS

The primary outcome of interest was the prevalent risk of MetS, a cluster of comorbid conditions including obesity, hypertension, lipid metabolism, and type 2 diabetes.^4^, ^23^ Five specific criteria from the laboratory results were used to assess for MetS, including abdominal obesity (waist circumference 90 cm or more in men, 80 cm or more in women), elevated blood pressure (130/85 mm Hg or higher), elevated fasting blood sugar (100 mg/dL or higher), high triglycerides (150 mg/dL or higher), and low high‐density lipoprotein (HDL) cholesterol (less than 40 mg/dL in men, less than 50 mg/dL in women).^4^, ^23^ We used a binary variable to indicate the prevalent risk of MetS. A two‐step approach was employed to address missing values. First, we excluded samples with more than two missing values out of the five specified conditions, as described in Figure 1. Second, individuals meeting three out of five conditions were classified as having MetS, while those with fewer than three conditions (including missing values) were classified as not having MetS.

#### 2.3.2 Area deprivation index and medical resource availability measures

We constructed an area‐level socioeconomic deprivation index (ADI) using principal component analysis of five measures in townships/districts.^24^ These measures included the proportions of low‐income households, individuals without a high school diploma, divorced and widowed individuals, the elderly population, and farming and fishing households. Two principal components were retained based on the covariance matrix eigenvalue, explaining 84% of the variance. Higher ADI scores indicate higher levels of deprivation. We further categorized ADI scores into terciles based on the 33rd and 67th percentiles among the 352 townships/districts in Taiwan.

For the area‐level medical resource availability index (MRI), four measures were used: the number of hospitals, the number of hospital beds, the number of clinics, and the number of physicians per 10,000 people. One principal component was retained, explaining 61.3% of the variance. Higher MRI scores indicate better medical resource availability. MRI scores were also categorized into terciles based on percentiles among 352 townships/districts in Taiwan. More detailed descriptions with respect to the ADI and MRI measures are listed in the supplemental files.

#### 2.3.3 Confounding covariates

Several variables that may affect outcomes were included as control variables. Demographic covariates included sex (male/female), age in years, age categories (<35, 35–44, 45–54, 55–64, 65+) education level (below middle school, above high school), marital status (single, divorced, or widowed/married), and self‐reported comorbid conditions (hypertension, diabetes mellites, hyperlipidemia). Health behavior covariates included smoking (yes/no), betel nut chewing (yes/no), and alcohol drinking (yes/no).

#### 2.3.4 Statistical approach

In addition to the principal component analysis used for constructing composite indexes, Student's *t*‐test and the chi‐square test were used to compare the means and proportions of demographic and outcome characteristics between subjects residing in the Cijin district and those in nationwide townships/districts other than Cijin. Given the high correlation among three key independent variables, we used univariable and three separate multivariable logistic regression models to examine the associations between ADI terciles, MRI terciles, or residing in the Cijin district, and the prevalent risk of MetS, respectively. In Model 1, we included the residential area variable, while Model 2 incorporated the ADI tercile variables, and Model 3 considered the MRI tercile variables. All models controlled for demographic and health behavior characteristics. Unadjusted odds ratios (OR), adjusted odds ratios (aOR) 95% confidence intervals, and *p*‐values were calculated. All statistical operations were performed using SAS version 9.4 (SAS institute, Cary, NC, USA). A *p*‐value of less than 0.05 was considered significant.

## 3 RESULTS

**FIGURE 2:**
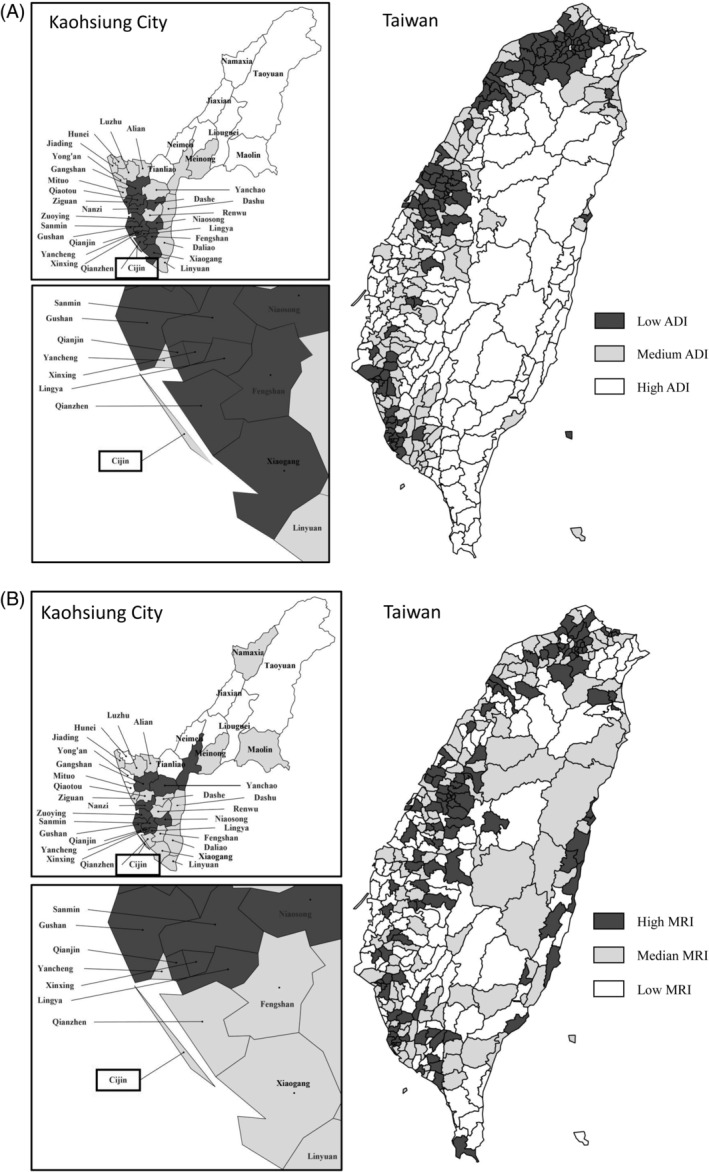
Geographic presentation for the tercile distribution of (A) area‐level socioeconomic deprivation index, ADI, and (B) medial resource index, MRI in Taiwan and Cijin townships/districts.

Table 1 displays the area‐level socioeconomic deprivation and medical resource availability measures, including ADI and MRI composite scores, for the 352 townships/districts and the Cijin district. The median ADI score was −0.18 (with a range between the 33rd and 67th percentiles of −0.8 and 0.45); the median MRI score was −0.69 (with a range between the 33rd and 67th percentiles of −0.93 and 0.12). The Cijin district ranked in the second tercile for both ADI and MRI among the 352 townships/districts in Taiwan. Figure 2 shows a geographic presentation of the tercile distribution of (A) ADI, and (B) MRI in 352 townships/ districts and the Cijin district in Taiwan.

Table 2 summaries study subjects' demographic characteristics and MetS conditions among subjects residing in the Cijin district and those in nationwide townships/districts other than Cijin. In Cijin, 37.76% of the subjects were male, averaging 49.97 years in age. In townships/districts other than Cijin, 36.96% of the subjects were male, averaging 49.03 years in age. Compared to the Cijin subjects, these subjects had significantly higher levels of education beyond high school (90.99%), a higher prevalence of hyperlipidemia (7.56%) and smoking (28.49%), but lower rates of hypertension (11.47%), diabetes (5.08%), and alcohol consumption (8.83%). Among overall nationwide townships/districts, 78.38% resided in a low ADI area, 18.93% in a median ADI area, and 2.68% in a high ADI area; 6.30%, 24.53%, and 69.18% resided in low, medium, and high MRI areas, respectively. Regarding MetS conditions and the associated risk, those in the Cijin district had a significantly increased risk of MetS (32.52% vs. 21.96%; *p*‐value <0.01), elevated blood pressure, and elevated fasting glucose levels (*p*‐value <0.01). Our results also demonstrated a moderately lower risk of abdominal obesity (*p*‐value = 0.042), with no significant difference in triglyceride and HDL cholesterol levels when compared to individuals residing in overall townships/districts.

**TABLE 2:** Demographic characteristics among study subjects in the Cijin district and nationwide township/districts in Taiwan, 2016–2020.

Variables	All study subjects	Subjects living in the Cijin district	Subjects living in nationwide township/districts other than the Cijin district^a^	*p*‐value
*N*	81,552	1430	80,122	
*Demographic characteristics*				
Sex (*N*, %)				0.533
Female	51,400 (63.03%)	890 (62.24%)	50,510 (63.04%)	
Male	30,152 (36.97%)	540 (37.76%)	29,612 (36.96%)	
Age in year (Mean ± sd)	49.05 ± 11.23	49.97 ± 15.41	49.03 ± 11.14	0.022
Age categories (*N*, %)				<0.001
<35	9546 (11.71%)	271 (18.95%)	9275 (11.58%)	
35–44	22,108 (27.11%)	261 (18.25%)	21,847 (27.27%)	
45–54	20,442 (25.07%)	314 (21.96%)	20,128 (25.12%)	
55–64	21,429 (26.28%)	331 (23.15%)	21,098 (26.33%)	
65+	8027 (9.84%)	253 (17.69%)	7774 (9.70%)	
Education level (*N*, %)				<0.001
Below middle school level	7821 (9.59%)	603 (42.17%)	7218 (9.01%)	
Above high school level	73,731 (90.41%)	827 (57.83%)	72,904 (90.99%)	
Marital status (*N*, %)				0.331
Unmarried, divorced, or widowed	23,918 (29.33%)	436 (30.49%)	23,482 (29.31%)	
Married	57,634 (70.67%)	994 (69.51%)	56,640 (70.69%)	
Comorbidity				
Hypertension (*N*, %)				<0.001
No	72,085 (88.39%)	1149 (80.35%)	70,936 (88.53%)	
Yes	9467 (11.61%)	281 (19.65%)	9186 (11.47%)	
Diabetes mellites (*N*, %)				<0.001
No	77,350 (94.85%)	1298 (90.77%)	76,052 (94.92%)	
Yes	4202 (5.15%)	132 (9.23%)	4070 (5.08%)	
Hyperlipidemia (*N*, %)				<0.001
No	75,437 (92.50%)	1373 (96.01%)	74,064 (92.44%)	
Yes	6115 (7.50%)	57 (3.99%)	6058 (7.56%)	
Smoking (*N*, %)				<0.001
No	58,491 (71.72%)	1195 (83.57%)	57,296 (71.51%)	
Yes	23,061 (28.28%)	235 (16.43%)	22,826 (28.49%)	
Betel nut chewing (*N*, %)				0.006
No	76,684 (94.03%)	1369 (95.73%)	75,315 (94.00%)	
Yes	4868 (5.97%)	61 (4.27%)	4807 (6.00%)	
Alcohol drinking (*N*, %)				0.017
No	74,328 (91.14%)	1278 (89.37%)	73,050 (91.17%)	
Yes	7224 (8.86%)	152 (10.63%)	7072 (8.83%)	
ADI tercile (*N*, %)^c^				<0.001
Low ADI	62,803 (77.01%)	‐	62,803 (78.38%)	
Median ADI	16,601 (20.36%)	1430 (100.00%)	15,171 (18.93%)	
High ADI	2148 (2.63%)	‐	2148 (2.68%)	
MRI tercile (*N*, %)^c^				<0.001
Low MRI	5045 (6.19%)	‐	5045 (6.30%)	
Median MRI	21,082 (25.85%)	1430 (100.00%)	19,652 (24.53%)	
High MRI	55,425 (67.96%)	‐	55,425 (69.18%)	
Metabolic syndrome conditions				
Abdominal obesity (male waist circumference > =90 cm; female waist circumference > =80 cm) (*N*, %)				
No	43,188 (52.96%)	795 (55.59%)	42,393 (52.91%)	0.042
Yes	38,326 (47.00%)	634 (44.34%)	37,692 (47.04%)	
Missing	38 (0.05%)	1 (0.07%)	37 (0.05%)	
High blood pressure (130/85 mm Hg or higher) (*N*, %)				
No	54,619 (66.97%)	673 (47.06%)	53,946 (67.33%)	<0.001
Yes	26,920 (33.01%)	757 (52.94%)	26,163 (32.65%)	
Missing	13 (0.02%)	0 (0.00%)	13 (0.02%)	
High fasting glucose (100 mg/dL or more) (*N*, %)				
No	64,303 (78.85%)	841 (58.81%)	63,462 (79.21%)	<0.001
Yes	17,206 (21.10%)	589 (41.19%)	16,617 (20.74%)	
Missing	43 (0.05%)	0 (0.00%)	43 (0.05%)	
High triglycerides (150 mg/dL or more) (*N*, %)				
No	63,873 (78.32%)	1107 (77.41%)	62,766 (78.39%)	0.373
Yes	17,636 (21.63%)	323 (22.59%)	17,313 (21.61%)	
Missing	43 (0.05%)	0 (0.00%)	43 (0.05%)	
Low HDL cholesterol (male less than 40 mg; female less than 50 mg/dL) (*N*, %)				
No	61,727 (75.69%)	1055 (73.78%)	60,672 (75.78%)	0.080
Yes	19,782 (24.26%)	375 (26.22%)	19,407 (24.22%)	
Missing	43 (0.05%)	0 (0.00%)	43 (0.05%)	
Presence for metabolic syndrome (*N*, %)^b^				
No	63,494 (77.86%)	965 (67.48%)	62,529 (78.04%)	<0.001
Yes	18,058 (22.14%)	465 (32.52%)	17,593 (21.96%)	

Abbreviations: ADI, area deprivation index; MRI, medical resource index.

^a^ Subjects living on the offshore islands, such as Kinmen and Matsu counties, were excluded.

^b^ Subjects who had at least three conditions of metabolic syndrome were classified into groups with metabolic syndrome.

^c^ The ADI and MRI measures in the Cijin district were only one‐point estimate. Therefore, there were no low or high ends of the ADI and MRI distributions.

Table 3 further presents the demographic, health behavior characteristic, and key independent variables between subjects with and without MetS conditions. All variables showed significant differences between subjects with and without Mets conditions. This study further presents both univariable and three multivariable logistic regression models in Table 4. In Model 1, we included the residential area variable, while Model 2 incorporated the ADI tercile variables, and Model 3 considered the MRI tercile variables. All models controlled for demographic and health behavior characteristics. Our findings indicate that after adjusting for these factors, residing in the Cijin district, characterized by relative socioeconomic deprivation and limited medical resource availability, was associated with significantly higher odds of prevalent risk of MetS (aOR = 1.45, 95% CI = 1.28–1.64, *p*‐value <0.001). Moreover, living in areas with medium (aOR = 1.12, 95% CI = 1.07–1.17, *p*‐value<0.001) and high ADI (aOR = 1.13, 95% CI = 1.01–1.25, *p*‐value <0.001) was also associated with a higher likelihood of MetS. Conversely, residing in high MRI areas was associated with a lower likelihood of prevalent risk of MetS (aOR = 0.92, 95% CI = 0.86–0.99, *p*‐value = 0.026). As for other confounding factors, male sex, older age, comorbid conditions (hypertension, diabetes, hyperlipidemia), smoking, and betel nut chewing were associated with a higher risk of prevalent MetS. Conversely, higher education levels and being married were associated with a lower risk of MetS.

**TABLE 3:** Demographic characteristics among study subjects between subjects with and without metabolic syndrome in Taiwan, 2016–2020.

Variables	All study subjects	Without metabolic syndrome	Metabolic syndrome^b^	*p‐*value
*N*	81,552	63,494	18,058
*Demographic characteristics*				
Sex (*N*, %)				
Female	51,400 (63.03%)	41,672 (65.63%)	9728 (53.87%)	<0.001
Male	30,152 (36.97%)	21,822 (34.37%)	8330 (46.13%)	
Age in year (Mean ± sd)	49.05 ± 11.23	48.03 ± 11.18	52.61 ± 10.68	
Age categories (*N*, %)				
<35	9546 (11.71%)	8505 (13.39%)	1041 (5.76%)	<0.001
35–44	22,108 (27.11%)	18,493 (29.13%)	3615 (20.02%)	
45–54	20,442 (25.07%)	15,780 (24.85%)	4662 (25.82%)	
55–64	21,429 (26.28%)	15,386 (24.23%)	6043 (33.46%)	
65+	8027 (9.84%)	5330 (8.39%)	2697 (14.94%)	
Education level (*N*, %)				
Below middle school level	7821 (9.59%)	5108 (8.04%)	2713 (15.02%)	<0.001
Above high school level	73,731 (90.41%)	58,386 (91.96%)	15,345 (84.98%)	
Marital status (*N*, %)				
Unmarried, divorced or widowed	23,918 (29.33%)	18,921 (29.80%)	4997 (27.67%)	<0.001
Married	57,634 (70.67%)	44,573 (70.20%)	13,061 (72.33%)	
Comorbidity				
Hypertension (*N*, %)				
No	72,085 (88.39%)	58,692 (92.44%)	13,393 (74.17%)	<0.001
Yes	9467 (11.61%)	4802 (7.56%)	4665 (25.83%)	
Diabetes mellites (*N*, %)				
No	77,350 (94.85%)	61,794 (97.32%)	15,556 (86.14%)	<0.001
Yes	4202 (5.15%)	1700 (2.68%)	2502 (13.86%)	
Hyperlipidemia (*N*, %)				
No	75,437 (92.50%)	60,095 (94.65%)	15,342 (84.96%)	<0.001
Yes	6115 (7.50%)	3399 (5.35%)	2716 (15.04%)	
Smoking (*N*, %)				
No	58,491 (71.72%)	46,786 (73.69%)	11,705 (64.82%)	<0.001
Yes	23,061 (28.28%)	16,708 (26.31%)	6353 (35.18%)	
Betel nut chewing (*N*, %)				
No	76,684 (94.03%)	60,444 (95.20%)	16,240 (89.93%)	<0.001
Yes	4868 (5.97%)	3050 (4.80%)	1818 (10.07%)	
Alcohol drinking (*N*, %)				
No	74,328 (91.14%)	58,436 (92.03%)	15,892 (88.01%)	<0.001
Yes	7224 (8.86%)	5058 (7.97%)	2166 (11.99%)	
*Key independent variables*				
Residential area (*N*, %)				
Live in township/districts other than the Cijin district^a^	80,122 (98.25%)	62,529 (98.48%)	17,593 (97.42%)	<0.001
Cijin district	1430 (1.75%)	965 (1.52%)	465 (2.58%)	
ADI tercile (*N*, %)				
Low ADI	62,803 (77.01%)	49,297 (77.64%)	13,506 (74.79%)	<0.001
Median ADI	16,601 (20.36%)	12,579 (19.81%)	4022 (22.27%)	
High ADI	2148 (2.63%)	1618 (2.55%)	530 (2.93%)	
MRI tercile (*N*, %)				
Low MRI	5045 (6.19%)	3872 (6.10%)	1173 (6.50%)	<0.001
Median MRI	21,082 (25.85%)	16,198 (25.51%)	4884 (27.05%)	
High MRI	55,425 (67.96%)	43,424 (68.39%)	12,001 (66.46%)	

Abbreviations: ADI, area deprivation index; MRI, medical resource index.

^a^ Subjects living on the offshore islands, such as Kinmen and Matsu counties, were excluded.

^b^ Subjects who had at least three conditions of metabolic syndrome were classified into groups with metabolic syndrome.

**TABLE 4:** Results from univariable and multivariable logistic regression models for the association between area‐level socioeconomic deprivation and medical resource availability and risk of metabolic syndrome, 2016–2020.

	Univariable analysis		Multivariable analysis					
			Model 1		Model 2		Model 3	
Variables	OR (95% CI)	*p*‐value	aOR (95% CI)	*p*‐value	aOR(95% CI)	*p*‐value	aOR (95% CI)	*p*‐value
Sex (Ref.: female)								
Male	1.64 (1.58–1.69)	<0.001	1.37 (1.32–1.43)	<0.001	1.38 (1.32–1.44)	<0.001	1.38 (1.32–1.44)	<0.001
Age categories (Ref.: <35)								
35–44	1.60 (1.48–1.72)	<0.001	1.55 (1.44–1.67)	<0.001	1.55 (1.43–1.67)	<0.001	1.54 (1.43–1.66)	<0.001
45–54	2.41 (2.25–2.59)	<0.001	2.02 (1.87–2.17)	<0.001	2.01 (1.87–2.17)	<0.001	2.01 (1.87–2.17)	<0.001
55–64	3.21 (2.99–3.44)	<0.001	2.22 (2.06–2.39)	<0.001	2.21 (2.05–2.38)	<0.001	2.21 (2.05–2.38)	<0.001
65+	4.13 (3.82–4.48)	<0.001	2.32 (2.12–2.52)	<0.001	2.33 (2.14–2.54)	<0.001	2.32 (2.13–2.53)	<0.001
Education level (Ref.: below middle school level)								
Above high school level	0.50 (0.47–0.52)	<0.001	0.71 (0.67–0.76)	<0.001	0.71 (0.67–0.75)	<0.001	0.70 (0.67–0.75)	<0.001
Marital status (Ref.: unmarried, divorced or widowed)								
Married	1.11 (1.07–1.15)	<0.001	0.90 (0.87–0.94)	<0.001	0.90 (0.87–0.94)	<0.001	0.90 (0.87–0.94)	<0.001
Comorbidities								
Hypertension (Ref.: No)								
Yes	4.26 (4.07–4.45)	<0.001	2.60 (2.48–2.74)	<0.001	2.61 (2.48–2.74)	<0.001	2.61 (2.49–2.74)	<0.001
Diabetes mellites (Ref.: No)								
Yes	5.85 (5.48–6.23)	<0.001	3.32 (3.09–3.56)	<0.001	3.32 (3.10–3.56)	<0.001	3.32 (3.10–3.56)	<0.001
Hyperlipidemia (Ref.: No)								
Yes	3.13 (2.97–3.30)	<0.001	1.52 (1.43–1.62)	<0.001	1.52 (1.43–1.61)	<0.001	1.52 (1.43–1.61)	<0.001
Smoking (Ref.: No)								
Yes	1.52 (1.47–1.58)	<0.001	1.13 (1.08–1.19)	<0.001	1.13 (1.08–1.19)	<0.001	1.13 (1.08–1.18)	<0.001
Betel nut chewing (Ref.: No)								
Yes	2.22 (2.09–2.36)	<0.001	1.38 (1.28–1.49)	<0.001	1.36 (1.27–1.47)	<0.001	1.37 (1.28–1.48)	<0.001
Alcohol drinking (Ref.: No)								
Yes	1.58 (1.49–1.66)	<0.001	1.03 (0.97–1.09)	0.403	1.03 (0.96–1.09)	0.417	1.03 (0.97–1.10)	0.354
Residential area (Ref.: township/districts; other than the Cijin district)								
Cijin district	1.71 (1.53–1.92)	<0.001	1.45 (1.28–1.64)	<0.001				
ADI tercile (Ref.: Low ADI)								
Median ADI	1.17 (1.12–1.22)	<0.001			1.12 (1.07–1.17)	<0.001		
High ADI	1.20 (1.08–1.32)	0.001			1.13 (1.01–1.25)	0.027		
MRI tercile (Ref.:Low MRI)								
Median MRI	1.00 (0.93–1.07)	0.899					1.01 (0.93–1.09)	0.903
High MRI	0.91 (0.85–0.98)	0.009					0.92 (0.86–0.99)	0.026

Abbreviations: ADI, area deprivation index; aOR, adjusted odds ratio; CI, confidence interval; MRI, medical resource index; OR, odds ratio; Ref., reference group.

## 4 DISCUSSION

This study investigated the association between socioeconomic area deprivation, the availability of medical resources in specific and relatively disadvantaged regions (such as the Cijin district), and other biological factors in relation to the prevalence of MetS. Our findings indicate that living in the Cijin district and in areas with higher levels of deprivation was significantly associated with an increased likelihood of prevalent risk of MetS. Conversely, residing in areas with higher levels of medical resources was associated with a reduced likelihood of prevalent risk of MetS.

Our study compared the Cijin district, characterized by median levels of both socioeconomic deprivation and medical resource availability, with nationwide townships/districts. We identified a higher prevalence of MetS in this specific district. To the best of our knowledge, this is the first study to investigate MetS prevalence and demographic characteristics in the Cijin district. Nearly half (42.17%) of the residents in the Cijin district have an educational attainment level below middle school and exhibit a higher prevalence of chronic diseases. Currently, the district's population exceeds 20,000 residents, with an aging population ratio higher than the Taiwan average, 19.5% compared to the national average of 17.8%.^21^ The district is serviced by only one small public hospital and a few clinics on the island, requiring residents to travel to the city for emergency care or treatment of moderate to severe illnesses. Public health must prioritize addressing healthcare access in areas with limited medical resources to improve early diagnosis and management of conditions like MetS. Tailoring strategies to the unique needs of such communities is essential for reducing healthcare disparities and enhancing overall health outcomes. Collaborative efforts between local authorities, healthcare providers, and community organizations are essential to ensure the effective implementation of these measures, ultimately leading to improved health outcomes in the Cijin district or similarly disadvantaged areas.

Consistent with prior studies, the current study demonstrates that area socioeconomic deprivation was associated with elevated prevalent risk of MetS.^12^, ^14^, ^17^, ^25^, ^26^ Our research indicates that approximately 22% of the population resides in areas with moderate to high levels of socioeconomic deprivation. These areas are predominantly situated in rural or mountainous regions in Taiwan (Figure 2A). The increased prevalence of MetS in these areas is the result of a complex interplay of factors, including restricted healthcare access, unhealthy lifestyle choices, resource limitations, social determinants of health, mental health challenges, and environmental influences.^11^, ^27^ For example, lower socioeconomic areas may have higher rates of unhealthy behaviors, such as poor dietary habits, sedentary lifestyles, and smoking and excessive alcohol consumption.^11^, ^27^ Addressing these disparities typically necessitates holistic public health and policy measures aimed at enhancing the overall well‐being of residents in these regions.

In socioeconomically deprived areas, residents may have limited access to healthcare facilities and services, including preventive care and health education. This can lead to delayed diagnosis and management of conditions like MetS.^11^, ^18^ Our research indicates that approximately 31% of the population resides in areas characterized by low to median levels of medical resources. Much like socioeconomically deprived areas, these regions are typically situated in rural and mountainous areas (Figure 1B). In our study, we investigated the availability of medical resources in various areas and its impact on the risk of MetS. The study results indicate a significant but weak association between high availability of medical resources and reduced likelihood of prevalent risk of MetS. This finding was consistent with prior studies by Farahani et al. (2019) and recent evidence from China and South Korea.^19^, ^28^ Several pivotal factors and policy interventions could mitigate the disadvantages faced by populations in areas with low to median levels of medical resources, such as enhancing medical resource availability, providing targeted intervention to underserved populations, and offering preventive care and early detection of diseases, which can significantly reduce the risk of MetS.

The current study independently examined the association between area deprivation, measured by the area‐level socioeconomic deprivation index, and medical resource availability, measured by the medical resource availability index, with the prevalence of MetS. Our findings suggest that unique socioeconomic factors captured by Taiwan's ADI may contribute to the increased risk of MetS in more deprived areas. In contrast, the relatively weak association between MRI and MetS suggests that medical resource availability may not be a significant factor in the incidence of MetS, with other socioeconomic conditions potentially playing a greater role. To reduce the prevalence of MetS in socioeconomically deprived areas, it is essential to address broader social and economic factors, rather than focusing solely on medical resource availability. Public health policies may play the key role in mitigating the risk of MetS in underserved communities.

Regarding other confounding factors, our study aligns with existing research, demonstrating that the occurrence of MetS is linked to various factors. These include biological factors such as male sex and older age, as well as physiological comorbid conditions such as hypertension, diabetes, and hyperlipidemia. Additionally, lifestyle factors like smoking, betel nut chewing, and alcohol intake are also associated with MetS. Conversely, higher education levels and being married were associated with a lower risk of MetS.^29^, ^30^, ^31^, ^32^, ^33^ To address the public health challenges revealed in this study, a comprehensive approach is needed. This should involve strategies to enhance educational opportunities, promote healthier lifestyles, and improve access to healthcare services, particularly for the aging population and those with chronic diseases.

This study has several limitations. First, we employed a cross‐sectional study design, which may limit our ability to establish causal inferences. This study could only examine prevalent cases of MetS due to the nature of data collection. Second, our use of laboratory results from physical examinations to establish MetS prevalence outcomes helped mitigate potential recall bias associated with self‐reported survey data. Third, both the ADI and MRI are based on aggregate data at the area level, which may not accurately reflect the socioeconomic or medical conditions of every individual within that area. Finally, participation in both the Taiwan Biobank and the Cijin study is voluntary, which could introduce self‐selection bias. Nevertheless, the strength of this study lies in its extensive national dataset and a representative sample from the Cijin district. This not only enhances its potential for generalizability but also offers valuable insights for shaping public health policies at both national and local levels in Taiwan.

## 5 CONCLUSION

This study found an association between socioeconomic area deprivation and the accessibility of medical resources, particularly in relatively disadvantaged regions such as the Cijin district. This linkage may contribute to the increased risk of MetS prevalence. In areas with socioeconomic disadvantages, residents often face challenges accessing healthcare facilities, preventive care, and health education. Effectively addressing these disparities requires comprehensive public health and policy interventions aimed at improving the overall well‐being of residents in these areas.

## CONFLICT OF INTEREST STATEMENT

We assure that each author meets authorship requirements. We declare that none of the authors has a conflict of interest with regard to this manuscript.

## ETHICS STATEMENT

This study was approved to use Taiwan biobank data from Academia Sinica (TWBR11103‐01) and ethical approval from the Institution Review Board (KMUHIRB‐E(I)‐20210300, KMUHIRB‐SV(I)‐20210082, and KMUHIRB‐G(I)‐20160025).

## Supporting information


**Data S1:** Supplementary Information

## Data Availability

The database application was approved by Taiwan biobank, Academia Sinica through its repository, and Kaohsiung Municipal Cijin Hospital. All data, including individual phenotypes of TWB participants, are available upon application for research purposes. A detailed description of TWB data availability and the application process can be found at https://taiwanview.twbiobank.org.tw/data_appl and https://www.biobank.org.tw/. In brief, investigators who are interested in obtaining the TWB data must submit an application that includes a detailed research proposal and an IRB approval from the applicant's home institution to the TWB Data Release Group (contact e‐mail: biobank@gate.sinica.edu.tw). The application will go through scientific and ethical reviews by external experts in the relevant scientific fields the Ethics and Governance Council and the Ethics and Governance Committee of TWB. Once approved, researchers will be able to obtain the data for the approved research projects during the approved time period. For researchers who are interested in applying but reside outside of Taiwan, or any cross‐country collaborations, an additional international data transfer agreement must be filed to the Ministry of Health and Welfare of Taiwan to enable sharing of the TWB individual‐level data or any of its derivatives.
