# Supplementary material for: Metabolic syndrome and spatial disparities: The role of socioeconomic deprivation and medical resource availability in the Cijin district, Taiwan
Source: Kaohsiung J Med Sci. 2024 Nov 14;40(12):1106–17. doi: 10.1002/kjm2.12908 (PMC11618489; doi:10.1002/kjm2.12908)
Supplement: Supplementary file 1 — Data S1: Supplementary Information [file KJM2-40-1106-s001.docx]

Supplementary Materials for:

“Metabolic Syndrome and Spatial Disparities: The Role of Socioeconomic Deprivation and Healthcare Access in the Cijin District, Taiwan”.

This supplementary material outlines the steps for constructing the Area-level Socioeconomic Deprivation Index (ADI) and the Medical Resource Availability Index (MRI) measures. Table A shows the data sources for constructing ADI and MRI.

Table A. Data source for constructing ADI and MRI

| **Table and Data source** | **Data sources** |
| --- | --- |
| **Area-level socioeconomic deprivation index (ADI)** |  |
| 1. District population statistics in 2010 | Ministry of the Interior (https://www.moi.gov.tw/) |
| 1. District population statistics in 2020 |  |
| 1. Agriculture, forestry, fisheries, and animal husbandry census statistics in 2010 |  |
| 1. Low-income household population statistics at district level in 2020 |  |
| 1. Population marital status statistics in 2020 |  |
| 1. Population educational attainment statistics in 2020 |  |
| **Medical Resource Availability Measures** |  |
| 1. Statistics in medical institutions and hospital beds in 2020 | Ministry of Health and Welfare  (<https://dep.mohw.gov.tw/>) |
| 1. Statistics in certified health professionals in acute hospitals in 2020 |  |
| 1. Statistics in certified health professionals in primary care clinics in 2020 |  |

1. Methods for constructing Area-level socioeconomic deprivation index (ADI)

We used publicly available government data to calculate the household proportions for the primary industry, the percentage of the population aged 65 years or older, the proportion of low-income households, the divorce and widowhood rates, and the percentage of the population with less than a high school education across different townships and districts. These five variables were then subjected to principal components analysis (PCA). The PCA results identified two principal components with eigenvalues greater than 1, explaining a total of 83% of the variance, with the first principal component accounting for 59% and the second for 24%. Prior to the analysis, the five variables were standardized using Z-scores. The principal component scores were calculated by multiplying the standardized variables by the eigenvectors associated with each principal component. The eigenvectors for the first principal component in relation to the five variables were 0.48, 0.34, 0.36, 0.52, and 0.52, respectively. Therefore, the first principal component score (PC1) for each township and district is calculated using the following formula:

PC1=0.48×Z_i1_+0.34×Z_i2_+0.36×Z_i3_+0.52×Z_i4_+0.52×Z_i5_

Where i was township/ districts)

This study identified two principal components with eigenvalues greater than one (as Table B shows), the following formula was used to calculate the area deprivation score:

ADI= PC1×($\frac{0.59}{0.83}$)+ PC2×($\frac{0.24}{0.83}$)

The area deprivation scores mentioned above will be categorized into three levels based on percentiles, from the lowest to the highest deprivation, corresponding to "low deprivation areas," "moderate deprivation areas," and "high deprivation areas," respectively.

Table B. Eigenvalues of the correlation matrix for the variables used to construct area-level socioeconomic deprivation index

| **Eigenvalues of the correlation matrix** | | | | |
| --- | --- | --- | --- | --- |
|  | Eigenvalues | Diff | Percent | Cumulative  Percent |
| **1** | 2.961 | 1.729 | 0.592 | 0.592 |
| **2** | 1.232 | 0.776 | 0.246 | 0.839 |
| **3** | 0.456 | 0.237 | 0.091 | 0.930 |
| **4** | 0.218 | 0.085 | 0.044 | 0.973 |
| **5** | 0.133 |  | 0.027 | 1.000 |

Principle Component


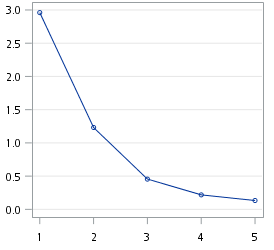


Eigenvalues

| **Eigenvector** | | | | | |
| --- | --- | --- | --- | --- | --- |
|  | **Prin1** | **Prin2** | **Prin3** | **Prin4** | **Prin5** |
| **lowincome_prop** | 0.335545 | 0.675186 | 0.371951 | 0.187544 | 0.507949 |
| **lowedu_prop** | 0.521122 | -0.158946 | -0.300647 | -0.702 | 0.346373 |
| **divorce_prop** | 0.520026 | -0.043637 | 0.55125 | -0.15244 | -0.632893 |
| **elder_prop** | 0.338191 | -0.681897 | 0.18286 | 0.505901 | 0.362311 |
| **industry_prop** | 0.480666 | 0.227973 | -0.658749 | 0.439145 | -0.300317 |

1. **Methods for constructing Medical Resource Availability Index (MRI) measures**

In this study, we used publicly available government data to conduct principal components analysis (PCA) on four variables for each township and district: the number of hospitals, the number of hospital beds, the number of clinics, and the number of physicians per 10,000 people. The analysis identified only one principal component with an eigenvalue greater than 1, which explained 61% of the variance (as Table C shows). Therefore, the first principal component score (PC1) represents the medical resource score. The medical resource score will be categorized into three levels based on percentiles, from areas with the least medical resources to those with the most, classified as "low medical resources," "moderate medical resources," and "high medical resources," respectively.

Table C. Eigenvalues of the correlation matrix for the variables used to construct area-level Medical Resource Availability Index (MRI) measures

| **Eigenvalues of the correlation matrix** | | | | |
| --- | --- | --- | --- | --- |
|  | Eigenvalues | Diff | Percent | Cumulative Percent |
| **1** | 2.452 | 1.651 | 0.613 | 0.613 |
| **2** | 0.801 | 0.195 | 0.200 | 0.813 |
| **3** | 0.606 | 0.465 | 0.152 | 0.965 |
| **4** | 0.141 |  | 0.035 | 1.000 |


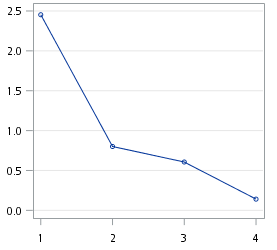


Principle Component

Eigenvalues

| **Eigenvector** | | | | |
| --- | --- | --- | --- | --- |
|  | **Prin1** | **Prin2** | **Prin3** | **Prin4** |
| **bed_prop** | 0.548151 | -0.498537 | 0.032091 | 0.670792 |
| **doctor_prop** | 0.54703 | -0.246534 | -0.523159 | -0.605214 |
| **clinics_prop** | 0.405308 | 0.81559 | -0.294932 | 0.289056 |
| **hospital_prop** | 0.485813 | 0.15967 | 0.798931 | -0.316545 |
